# Constitutive Activation of PrfA Tilts the Balance of *Listeria monocytogenes* Fitness Towards Life within the Host versus Environmental Survival

**DOI:** 10.1371/journal.pone.0015138

**Published:** 2010-12-07

**Authors:** Joseph C. Bruno, Nancy E. Freitag

**Affiliations:** 1 Department of Global Health, University of Washington, Seattle, Washington, United States of America; 2 Department of Microbiology and Immunology, University of Illinois at Chicago, Chicago, Illinois, United States of America; East Carolina University School of Medicine, United States of America

## Abstract

PrfA is a key regulator of *Listeria monocytogenes* pathogenesis and induces the expression of multiple virulence factors within the infected host. PrfA is post-translationally regulated such that the protein becomes activated upon bacterial entry into the cell cytosol. The signal that triggers PrfA activation remains unknown, however mutations have been identified (prfA* mutations) that lock the protein into a high activity state. In this report we examine the consequences of constitutive PrfA activation on *L. monocytogenes* fitness both *in vitro* and *in vivo*. Whereas prfA* mutants were hyper-virulent during animal infection, the mutants were compromised for fitness in broth culture and under conditions of stress. Broth culture prfA*-associated fitness defects were alleviated when glycerol was provided as the principal carbon source; under these conditions prfA* mutants exhibited a competitive advantage over wild type strains. Glycerol and other three carbon sugars have been reported to serve as primary carbon sources for *L. monocytogenes* during cytosolic growth, thus prfA* mutants are metabolically-primed for replication within eukaryotic cells. These results indicate the critical need for environment-appropriate regulation of PrfA activity to enable *L. monocytogenes* to optimize bacterial fitness inside and outside of host cells.

## Introduction

The environmental bacterial pathogen *Listeria monocytogenes* is an intriguing example of a microorganism that has become well adapted to life in the soil as well as to life within the cytosol of mammalian host cells. This bacterium is widespread in the environment where it is believed to live as a saprophyte on decaying plant material [1]. Upon ingestion by a susceptible mammalian host, *L. monocytogenes* transitions into a physiological state that facilitates bacterial survival and replication within host cells [2], [3]. While disease caused by *L. monocytogenes* in healthy individuals is usually restricted to a self-limiting gastroenteritis, in immunocompromised individuals and pregnant women *L. monocytogenes* is capable of causing systemic infections that lead to meningitis, encephalitis, and in the case of pregnant women, infection of the developing fetus leading to abortion, stillbirth, or neonatal infections [4], [5]. *L. monocytogenes* contamination of food products has resulted in some of the most expensive food recalls in U.S. history [2], [6]–[12] and this is thought to reflect the bacterium's widespread environmental distribution and its ability to withstand a variety of stress conditions [13]–[16].

A significant amount of research has focused on the mechanisms used by *L. monocytogenes* to establish its replication niche within mammalian host cells. *L. monocytogenes* invades a wide variety of cell types and is capable of escaping from the phagosome following cell entry, of replicating within the cytosol, and of utilizing host cell actin polymerization machinery to propel itself through the cytosol and into neighboring cells [3], [5], [17]. To survive and flourish within eukaryotic cells the bacterium requires the regulated expression of a number of secreted virulence factors, and the expression of most of these gene products is regulated by a transcriptional regulator known as PrfA [18]. PrfA is an essential regulator of *L. monocytogenes* pathogenesis, and bacterial mutants that lack functional PrfA are severely attenuated in animal infection models [19], [20].

PrfA is a member of the Crp/Fnr family of transcriptional activators, and members of this family appear to require post-translational modification or the binding of a small molecule co-factor for full activity [21]–[23]. PrfA activation occurs upon bacterial entry into the host cell cytosol and is required for the increased expression of gene products that promote bacterial cell-to-cell spread [19], [24]–[29]. *L. monocytogenes* strains that encode a mutant form of *prfA* (*prfA* Y154C) whose product fails to become activated following cytosol entry are severely attenuated for virulence [30]. The signal that induces PrfA activation remains unknown, however *L. monocytogenes* strains have been isolated that contain mutations within *prfA* resulting in constitutive PrfA activation (prfA* alleles) [31]–[36]. prfA* strains exhibit enhanced invasion of host cells, rapid escape from the phagosome, and an apparent increase virulence following intravenous injection of mice [32], [37]. In broth culture prfA* mutants exhibit the same high levels of PrfA-dependent gene expression normally observed for bacteria during intracellular growth [32], [38]–[40]. While a number of mutations have been identified in *prfA* that confer activation, the absolute level of activation observed for different amino acid substitution mutants can vary, with mutants exhibiting the highest level of activation most closely resembling the levels of activation observed for cytosolic bacteria [32]–[34], [41], [42].

Given that *L. monocytogenes* has evolved specific mechanisms to regulate PrfA activity in response to environmental conditions found inside and outside of host cells, we sought to determine the impact of constitutive PrfA activation on the fitness of *L. monocytogenes* by comparing the growth of strains in broth culture and in tissue culture and mouse infection models. Our results indicate that PrfA activity must be carefully modulated in response to environmental signals so as to enable *L. monocytogenes* to optimize bacterial fitness both inside and outside of the infected host.

## Results

### Constitutive activation of PrfA reduces the fitness of *L. monocytogenes* in nutrient-rich broth

Until recently, it has proven difficult to construct isogenic L. monocytogenes prfA* mutant strains containing the alleles that confer the highest PrfA activity by standard methods. As a result, these high activity prfA* mutations have been introduced into Δ*prfA* strains on plasmids [30], [31], [34], [40], [43], [44]. While these approaches have been informative, there are associated caveats that include multicopy plasmid effects or altered gene expression profiles resulting from the use of integrated plasmids in ectopic locations. We recently reported the successful construction of high activity prfA* isogenic mutants in strains containing promoterless copies of the genes encoding β-glucuronidase (*gus*) and neomycin resistance (*neo*) located in the chromosome downstream of the PrfA-dependent gene *actA* [41]. Isogenic prfA* mutants constructed via allelic exchange were isolated based on the PrfA*-dependent increase in *actA-gus-neo* expression that enabled selection for prfA* colonies on selective media containing neomycin and 5-bromo-4-chloro-3-indolyl-β-D-glucuronic acid (x-gluc), a substrate for GUS activity. This approach now enables the direct comparison of independently isolated L. monocytogenes prfA* mutants with strains containing the wild type allele.

**Figure 1:**
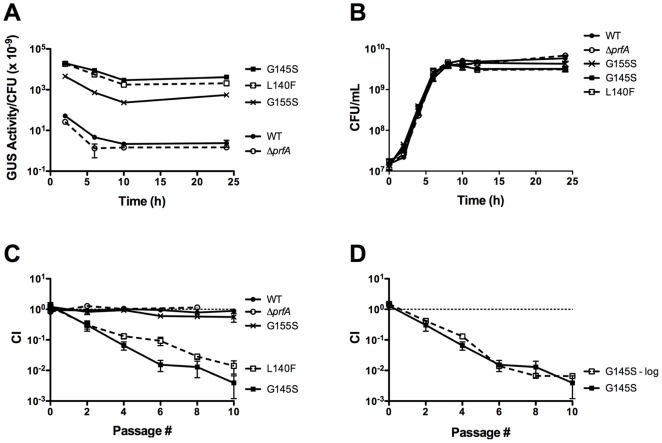
prfA* mutants exhibit a competitive defect when grown with wild type in nutrient rich broth. (**A**) Comparison of levels of PrfA activation between different prfA* mutant strains as measured by *actA* expression. PrfA-dependent *actA* expression levels were measured by monitoring the GUS activity of *L. monocytogenes* strains containing an *actA-gus* transcriptional fusion. Bacteria were grown in BHI at 37°C with shaking, and units of GUS activity were normalized to CFU/mL. Each datum point represents the mean ± standard deviation of a GUS assay measured in duplicate, and each GUS activity profile is representative of 2 independent experiments. The prfA* mutants are referred to by their PrfA amino acid mutations. (**B**) Monoculture growth of wild type, Δ*prfA*, and prfA* L. monocytogenes strains in BHI at 37°C with shaking. Each growth curve is representative of two independent experiments. (**C**) prfA*mutants exhibit a competitive defect when grown with wild type *L. monocytogenes.* Wild type *L. monocytogenes* was transformed with the integrative plasmid vector pPL2 to confer chloramphenicol resistance, and then assessed for growth in BHI at 37°C in the presence of chloramphenicol-sensitive test strains as indicated. Mixed cultures were subjected to repeated cycles of culture dilution and outgrowth every 24 hours into fresh BHI. The competitive index (CI) values of the mixed cultures were determined immediately prior to each dilution as described in Experimental Procedures. The data represent the means ± standard errors of three independent experiments. (**D**) The competitive defect of prfA* strains occurs during logarithmic growth. A mixed culture of the *prfA* G145S mutant and the wild type strain was subjected to repeated cycles of culture dilution and outgrowth at late-log phase (OD_600_ of 0.8–1.0≈8×10^8^–1×10^9^ CFU/mL) (indicated at ‘G145S - log’). CI values were determined immediately prior to each dilution. The data represent the means ± standard errors of two independent experiments.

To assess if the constitutive activation of PrfA influences the fitness of *L. monocytogenes* outside of host cells, strains containing mid-level (*prfA* G155S) or high-level (*prfA* G145S and *prfA* L140F) prfA* activity mutations [41] were compared with a wild type strain for growth in BHI broth. Consistent with previous reports, prfA* mutations conferred high levels of PrfA activity in broth culture as indicated by *actA* expression levels (Fig. 1A). Expression from the *actA* promoter for the *prfA* G155S and *prfA* G145S mutants was 230-fold and 1870-fold higher respectively than the levels observed for wild type *prfA* strains after 24 hours of growth in BHI (Fig. 1A). Overall growth of the prfA* mutants was very similar to that of the wild type strain, although the doubling times of the prfA* mutants during logarithmic growth were slightly longer (Table S1) and the final bacterial cell densities at stationary phase were slightly lower in the prfA* monocultures than in the wild type monocultures (Fig. 1B).

In contrast to monoculture growth, pronounced fitness effects were observed for high activity prfA* strains when the mutants were mixed and grown with wild type bacteria in BHI. Each prfA* mutant exhibited a competitive defect when cultures were inoculated in equal numbers with wild type bacteria and grown to stationary phase with subsequent cycles of dilution and outgrowth (Fig. 1C). After nine sequential cycles of overnight growth and dilution, wild type bacteria were observed in two-fold greater numbers in comparison to the mid-level *prfA* G155S mutant and 200-fold greater numbers in comparison to the high-level *prfA* G145S and *prfA* L140F mutant strains (Fig. 1C). A direct correlation thus appeared to exist between the level of PrfA activation conferred by a prfA* mutation and the magnitude of the competitive defect observed in broth culture. In addition, the identical phenotypes observed for independently derived *prfA* G145S and *prfA* L140F strains confirmed that the observed defect resulted from the mutational activation of *prfA* and did not reflect a second site mutation; an additional independently derived prfA* mutant (*prfA* Y63C) likewise exhibited an identical competitive defect (J. Bruno, unpublished). Bacterial supernatants derived from wild type cultures did not inhibit the growth of prfA* mutants (J. Bruno, unpublished), indicating that there is no apparent inhibitory substance produced by wild type bacteria that compromised mutant growth.

To determine if the competitive defects exhibited by the prfA* mutants occurred during logarithmic growth or whether the defects were associated with entry into or survival during stationary phase, mixed cultures were diluted into fresh BHI upon reaching late-logarithmic phase (OD_600_ of 0.8–1.0), prior to bacterial entry into stationary phase. When mixed cultures of wild type and *prfA* G145S bacteria were grown under these conditions, the resulting competitive defect was essentially identical to the competitive defect observed for mixed cultures grown to stationary phase (Fig. 1D). This indicates that constitutive activation of PrfA impairs the competitive fitness of *L. monocytogenes* in broth culture during logarithmic growth.

#### The presence of glucose exacerbates the competitive defect exhibited by L. monocytogenes prfA* strains

**Figure 2:**
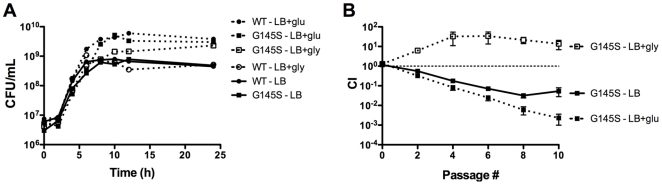
Effects of different carbon sources on monoculture growth and competitive index of a prfA* mutant. (**A**) Growth curves of the wild type and *prfA* G145S *L. monocytogenes* strains in buffered LB (pH 7.4) with and without 55 mM of either glucose (glu) or glycerol (gly) at 37°C with shaking were determined by measuring CFU/mL at the specified time points. Each growth curve is representative of two independent experiments. (**B**) The wild type cam^R^ strain was mixed with the chloramphenicol-sensitive *prfA* G145S mutant in buffered LB with and without 55 mM of either glucose (glu) or glycerol (gly) at 37°C with shaking. Mixed cultures were subjected to repeated cycles of growth and dilution (1∶100) into fresh media every 24 hours. CI values were determined immediately prior to each dilution. The data represent the means ± standard errors of three independent experiments.

It has been previously reported that multicopy plasmid-based over-expression of constitutively activated PrfA (*prfA* G145S) interferes with bacterial utilization of glucose as a carbon source [43], [44]. To examine if isogenic prfA* mutants exhibited a fitness defect in the presence of glucose, the *prfA* G145S mutant was grown in LB buffered to pH 7.4 and supplemented with 55 mM of glucose. LB was selected for monitoring growth as *L. monocytogenes* requires an added carbon source for optimal growth in this medium. Similar to the observations made for prfA* monocultures in BHI, cultures grown in LB and glucose-supplemented LB resembled the wild type strain with only subtle growth differences, indicating that the isogenic prfA* strains were able to efficiently use glucose as a carbon source (Fig. 2A and Table S1). However, when *prfA* G145S cultures were mixed with the wild type strain and grown in LB or in LB supplemented with glucose, the competitive defect exhibited by the *prfA* G145S mutant in LB with glucose was of greater magnitude than that exhibited in LB alone (Fig. 2B). After seven cycles of dilution and outgrowth, wild type bacteria outnumbered the *prfA* G145S mutants by 30-fold and 170-fold in LB and in glucose-supplemented LB, respectively (Fig. 2B). The presence of glucose thus exacerbated the competitive defect associated with PrfA activation in broth culture.

### Constitutive activation of PrfA increases the fitness of *L. monocytogenes* in the presence of glycerol

Stoll *et al.* have reported that plasmid-based over-expression of prfA* decreased the fitness of *L. monocytogenes* in the presence of glycerol based on reduced growth in media where glycerol was the main or sole carbon source [44]. To determine if isogenic prfA* mutants were compromised for growth in the presence of glycerol, the *prfA* G145S mutant was grown in LB buffered to pH 7.4 and supplemented with 55 mM glycerol. Surprisingly, monocultures of the *prfA* G145S mutant in glycerol-supplemented LB grew to five-fold higher cell densities in comparison to wild type strains (Fig. 2A). Consistent with this growth advantage, the *prfA* G145S mutant exhibited a competitive advantage when it was mixed and grown with wild type *L. monocytogenes* in glycerol-supplemented LB. After seven cycles of dilution and outgrowth, *prfA* G145S outnumbered wild type bacteria by more than 20-fold (Fig. 2B). These findings indicate that constitutive activation of PrfA increases the fitness of *L. monocytogenes* in the presence of glycerol. The findings further indicate that competitive defects associated with the prfA* strains in other media cannot simply be attributed to the metabolic burden of increased PrfA-dependent gene product expression, as high expression levels are maintained by prfA* strains in the presence of glycerol ([44], [45] and J. Bruno, unpublished).

### Constitutive activation of PrfA increases the sensitivity of *L. monocytogenes* to osmotic stress and acid stress

**Figure 3:**
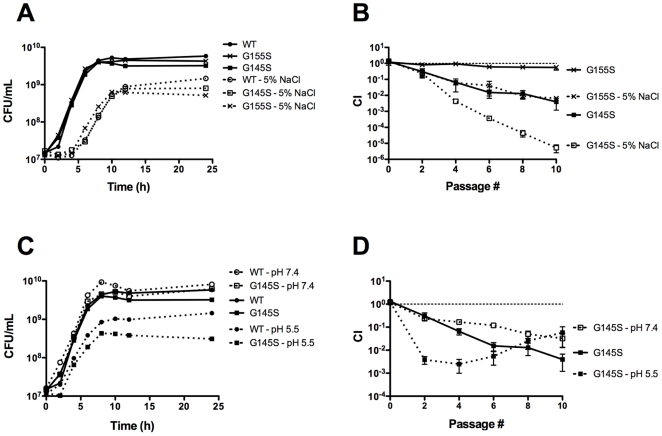
Stress conditions exacerbate the competitive defects exhibited by prfA* mutants. (**A**) Monoculture growth curves of *L. monocytogenes* strains in BHI supplemented with 5% NaCl at 37°C were determined by measuring CFU/mL at the specified time points. The growth curves of wild type and *prfA* G145S *L. monocytogenes* in BHI without additional NaCl are included. (**B**) Competitive index of wild type cam^R^ strain mixed with a chloramphenicol-sensitive prfA* mutant in BHI supplemented with 5% NaCl at 37°C. Mixed cultures were subjected to repeated cycles of growth and dilution (1∶100) into fresh media every 24 hours. CI values were determined immediately prior to each passage. The data represent the means ± standard errors of three independent experiments. (**C**) Monoculture growth curves of *L. monocytogenes* strains in BHI buffered to pH 7.4 or pH 5.5 at 37°C were determined by measuring CFU/mL at the specified time points. (**D**) Competitive index of the wild type cam^R^ strain mixed with a chloramphenicol-sensitive prfA* mutant in BHI buffered to pH 7.4 or 5.5 at 37°C. Mixed cultures were subjected to repeated cycles of growth and dilution (1∶100) into fresh media every 24 hours. CI values were determined immediately prior to each dilution. The data represent the means ± standard errors of three independent experiments.

The ability of *L. monocytogenes* to withstand a variety of stresses is vital for its survival and replication in disparate environments [5], [13], [46], [47], including food processing facilities [9], [48] and the gastrointestinal tract [3], [17], [49], [50]. To determine if constitutive activation of PrfA influences the ability of *L. monocytogenes* to respond to stress, monoculture and mixed culture growth of the prfA* mutants and wild type *L. monocytogenes* was examined under two different stress conditions, osmotic stress and acid stress. Although no dramatic differences were observed for mutant and wild type strains with respect to growth in monoculture (Fig. 3AC), the *prfA* G155S mutant and the *prfA* G145S mutant exhibited more severe competitive defects when mixed with the wild type strain and grown in BHI supplemented with 5% NaCl in comparison to BHI lacking additional NaCl (Fig. 3B). After nine cycles of dilution and outgrowth, wild type bacteria outnumbered mutants by more than 150-fold (*prfA* G155S) and 200,000-fold (*prfA* G145S) in the presence of additional NaCl, in comparison to differences of 2-fold (*prfA* G155S) and 200-fold (*prfA* G145S) in growth media lacking added NaCl (Fig. 3B).

The *prfA* G145S mutant exhibited a similarly exacerbated competitive defect under acid stress. When *L. monocytogenes* was grown in unbuffered BHI broth at 37°C, the pH was observed to decrease from approximately 7.2 to 6.0 after 24 hours of growth (J. Bruno, unpublished). When grown with wild type *L. monocytogenes* strains, the *prfA* G145S mutant initially exhibited a more severe competitive defect in BHI buffered to pH 5.5 than in unbuffered BHI. After three cycles of dilution and outgrowth, wild type bacteria were present in 400-fold greater numbers than the *prfA* G145S mutant in BHI pH 5.5 in comparison to 15-fold greater numbers in unbuffered BHI (Fig. 3D). Interestingly, the large competitive defect exhibited by the *prfA* G145S mutant during the first three cycles of dilution and outgrowth in BHI pH 5.5 shifted to a competitive advantage with subsequent cycles, reducing the wild type advantage from 400-fold to 20-fold after nine cycles (Fig. 3D). This ratio was similar to the ratio observed after nine cycles of dilution and outgrowth in BHI buffered to pH 7.4 (Fig. 3D). These findings suggest that the *prfA* G145S mutant goes through an adaptation or acid tolerance response [51] that increases its tolerance to acid stress to wild-type levels or even beyond. Overall, these findings indicate that constitutive activation of PrfA impaired the ability of *L. monocytogenes* to respond to osmotic stress as well as its initial response to acid stress conditions.

### The impaired stress response of prfA* mutants does not result from impaired function of the stress-associated sigma factor, SigB

**Figure 4:**
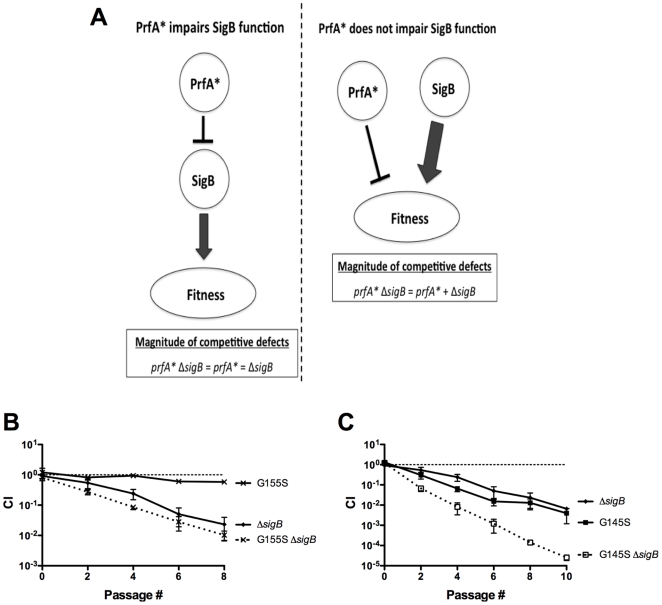
The increased susceptibility of prfA* cultures to stress is unrelated to *sigB* function. (**A**) Rationale regarding how the competitive defect exhibited by a prfA* Δ*sigB* double mutant can be used to determine if the competitive defect associated with constitutive PrfA activation is related to an impairment of SigB function. If constitutive activation of PrfA (PrfA*) impairs SigB function, the magnitude of the competitive defect exhibited by a prfA* Δ*sigB* double mutant strain will be equivalent to the magnitude of the competitive defects exhibited by the prfA* and Δ*sigB* single mutants. If constitutive activation of PrfA (PrfA*) does not impair SigB function, the magnitude of the competitive defect exhibited by a prfA* Δ*sigB* double mutant will be equivalent to the sum of the magnitudes of the competitive defects exhibited by the prfA* and Δ*sigB* single mutants. (**B**) Assessment of the competitive index for the *prfA* G155S Δ*sigB* double mutant. The wild type cam^R^ strain was mixed with a chloramphenicol-sensitive test strain in BHI at 37°C. Mixed cultures were subjected to repeated cycles of growth and dilution (1∶100) into fresh BHI every 24 hours, and CI values were determined immediately prior to each dilution. The data represent the means ± standard errors of two independent experiments. (**C**) Assessment of the competitive index for the *prfA* G145S Δ*sigB* double mutant. The wild type cam^R^ strain was mixed with a chloramphenicol-sensitive test strain in BHI at 37°C. Mixed cultures were subjected to repeated cycles of growth and dilution (1∶100) into fresh BHI every 24 hours, and CI values were determined immediately prior to each dilution. The data represent the means ± standard errors of two independent experiments.

The exacerbated decrease in the bacterial fitness of prfA* mutants when subjected to two different stress conditions suggested that a general response related to stress tolerance may be compromised by constitutive activation of PrfA. A central regulatory component that contributes to the ability of *L. monocytogenes* to survive various stress conditions is the alternative RNA polymerase sigma factor SigB [16], [52]. SigB contributes to *prfA* expression [25], and several previous studies have suggested the existence of functional overlap between SigB and PrfA in regulating the expression of *L. monocytogenes* genes that contribute to virulence and/or stress response [40], [53]–[60]. While Δ*sigB* growth in monoculture resembled that of the wild type strain (Supplemental Fig. S2), Δ*sigB* mutants exhibited a competitive defect when mixed with the wild type strain in BHI, indicating that loss of SigB function decreases the competitive fitness of *L. monocytogenes* (Fig. 4). Interestingly, the magnitude of the competitive defect exhibited by the Δ*sigB* mutant closely resembled that observed for *prfA* G145S mutants in BHI (Fig. 4C).

To determine if the competitive defect associated with prfA* strains was related to an impairment of SigB function, *prfA* G155S Δ*sigB* and *prfA* G145S Δ*sigB* double mutants were tested in broth competition assays. If PrfA* impairs SigB function, one would anticipate that the magnitude of the competitive defect exhibited by a prfA* Δ*sigB* double mutant would be equivalent to that exhibited by either single mutant (Fig. 4A). If however the magnitude of the competitive defect exhibited by a prfA* Δ*sigB* double mutant was equivalent to the sum of the defects exhibited by the prfA* and Δ*sigB* single mutants (Fig. 4A), this would suggest that PrfA and SigB alter stress resistance through separate pathways. The competitive defect of a prfA* Δ*sigB* double mutant was found to be equivalent to the sum of the defects of the prfA* and the Δ*sigB* single mutants, and the additive effect of prfA* and Δ*sigB* in the double mutant strain was evident throughout the course of mixed growth (Fig. 4BC). Therefore, the stress related competitive defect associated with the constitutive activation of PrfA appears distinct from the defect associated with the loss of SigB function.

### Constitutive activation of PrfA enhances *L. monocytogenes* virulence following intravenous and intragastric infection of mice

**Figure 5:**
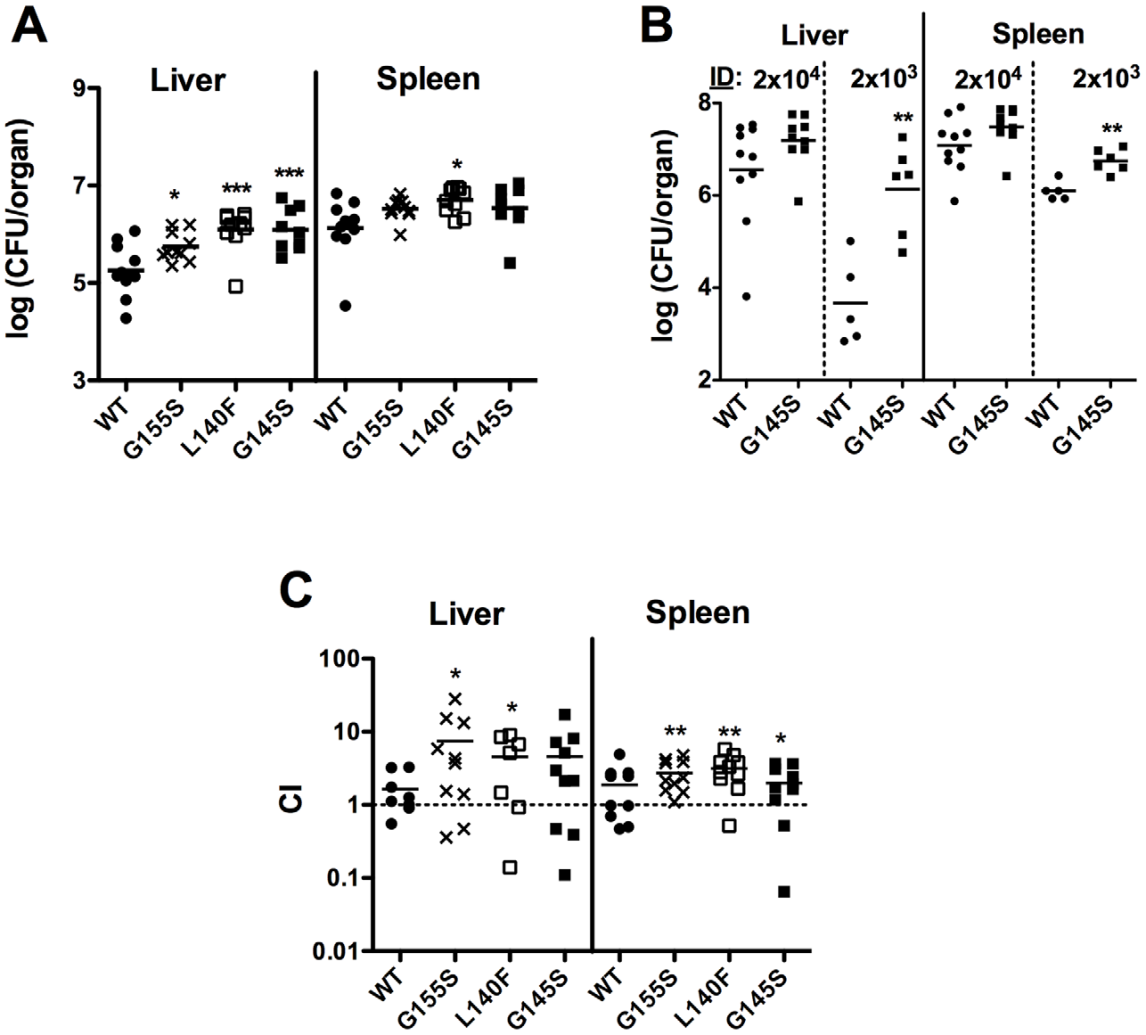
Growth of the prfA* mutants in the livers and spleens of intravenously infected mice. 7–8 week old ND4 Swiss Webster mice were infected with *L. monocytogenes* via tail-vein injections, and at the specified times post-infection (pi), the bacterial loads of the livers and spleens were determined as described in Experimental Procedures. Data are presented as scatter dot plots, with horizontal bars representing means. (**A**) Infection of mice with 2×10^4^ CFU wild type, *prfA* G155S, *prfA* L140F, or *prfA* G145S mutants. Organs were harvested 24 hours pi. Asterisks denote statistically significant differences between the amounts of prfA* mutant and wild type CFU recovered using a one-way analysis of variance with Dunnett's post-test (*, P<0.05; ***, P<0.001). (**B**) Comparison of infection with 2×10^3^ or 2×10^4^ CFU of wild type and *prfA* G145S mutant. Organs were harvested 48 hours pi. Asterisks denote statistically significant differences between the amounts of *prfA* G145S mutant and wild type CFU recovered using an unpaired t test with a two-tailed P value (**, P<0.01). (**C**) Competitive index of wild type and prfA* strains. Prior to intravenous injection, the wild type Erm^r^ reference strain and the indicated test strain were mixed 1∶1 for a total bacterial suspension of 2×10^4^ CFU. For each organ, the competitive index (CI) value (CI =  test strain CFU/reference strain CFU) was determined as described in Experimental Procedures. Asterisks denote statistically significant CI values compared to 1 using a one-sample t test with a two-tailed P value (*, P<0.05; **, P<0.01).

Previous studies have reported that the prfA* mutants with mid-level PrfA activity (*prfA* G155S mutants) were fully virulent when intravenously inoculated into mice based on the bacterial CFU required for a 50% lethal dose (LD_50_) [32]. Consistent with this observation, mice intravenously infected with 2×10^4^ CFU had significantly higher numbers of prfA* bacteria (*prfA* G155S, *prfA* G145S, and *prfA* L140F) recovered from the livers and spleens at 24 hours post-infection compared to those infected with wild type bacteria (Fig. 5A) (the liver and spleen are the primary organ targets for *L. monocytogenes* replication [5]). Although the difference was not statistically significant at 48 hours post-infection, the bacterial burdens of the livers and spleens from mice infected with the prfA* mutant tended to be higher than in organs associated with wild type infection (Fig. 5B and J. Bruno, unpublished). The hyper-virulent phenotype of the prfA* mutants was more apparent when the infectious dose was reduced by ten-fold to 2×10^3^ CFU; the bacterial burdens of the livers and spleens from mice infected with the *prfA* G145S mutant at 48 hours post-infection were approximately 300-fold and 4.5-fold higher in liver and spleen than those of mice infected with wild type bacteria (P<0.01 for both organs) (Fig. 5B). In mixed infection, the prfA* mutants consistently exhibited a competitive advantage over wild type strains (Fig. 5C). The competitive index values determined for each liver and spleen for intravenously infected mice at 48 hours post-infection showed that, on average, 2- to 7-fold more prfA* bacteria were recovered from each organ in comparison to wild type (Fig. 5C).

**Figure 6:**
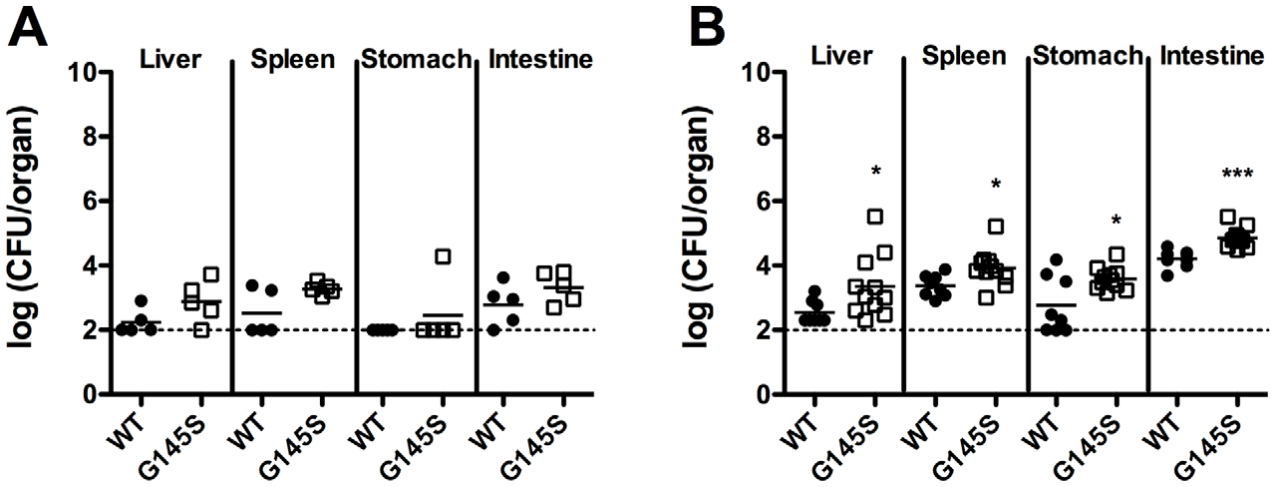
Growth of wild type *prfA inlA^m^* and *prfA* G145S *inlA^m^* strains in intragastrically infected mice. 8–10 week old C57BL/6 mice were infected with (**A**) 5×10^7^ CFU or (**B**) 5×10^9^ CFU *L. monocytogenes* via intragastric injection and 72 hours post-infection the bacterial loads of the livers, spleens, stomachs, and intestines were determined as described in Experimental Procedures. Data are presented as scatter dot plots, with horizontal bars representing the means. The log (CFU/organ) value of 1.996 is the limit of detection and is represented by a dashed line. A datum point on the dashed line represents an organ from which no detectable CFU were obtained. Asterisks denote statistically significant differences between the amounts of *prfA* G145S mutant and wild type CFU recovered using an unpaired t test with a two-tailed P value (*, P<0.05; ***, P<0.001).

Although constitutive activation of PrfA enhanced bacterial infection following intravenous injection of mice, the increased sensitivity to both osmotic stress and acid stress observed for the mutant strains (Fig. 3) suggested that the virulence of the prfA* mutants might be attenuated if administered orally, the more natural route of infection. We therefore examined the consequences of constitutive PrfA activation on the fitness of *L. monocytogenes* within an animal host following intragastric inoculation. Intragastric infection with either the *prfA* G145S mutant or wild type *L. monocytogenes* strain was carried out following the introduction of the *inlA^m^* mutation into each strain background to enhance bacterial interaction with mouse E-cadherin and translocation of bacteria across the intestinal epithelium [61]. Surprisingly, 2- to 7-fold more bacteria were recovered from the livers, spleens, stomachs, and intestines of mice infected with the *prfA* G145S *inlA^m^* mutant than from the organs of mice infected with the wild type *prfA inlA^m^* strain at infectious doses of either 5×10^7^ CFU or 5×10^9^ CFU (Fig. 6). These findings indicate that constitutive activation of PrfA enhances the fitness of *L. monocytogenes* inside of the host following either intravenous or intragastric inoculation.

## Discussion

Central to the ability of *L. monocytogenes* to flourish in a wide variety of environments is the appropriate expression of gene products that facilitate bacterial survival and replication within a given niche. *L. monocytogenes* occupies disparate environments that range from soil and food-processing plants to the gastrointestinal tract and cell cytosol of infected mammals [3], [5], [9], [13], [17], [46]–[50]. It has been previously demonstrated that dramatic increases in PrfA activity and PrfA-dependent gene expression occur following entry of *L. monocytogenes* into the cytosol [18], [42], [62], [63]. We therefore sought to investigate the importance of appropriate regulation of PrfA activity under different environmental conditions in the context of *L. monocytogenes* fitness inside and outside of infected host cells. Our results indicate that while constitutive activation of PrfA serves to enhance bacterial virulence within the infected host, in most cases PrfA activation decreases bacterial fitness outside of host cells. *L. monocytogenes* therefore regulates PrfA activity so as to optimally balance life in the outside environment with life inside of the host.

Environmental regulation of PrfA activity suggests that the high levels of PrfA activity required for intracellular life are detrimental to the fitness of *L. monocytogenes* outside of a host cell, and limited analyses of *L. monocytogenes* field strains appear to support this hypothesis. Although far from being exhaustively examined, no field strain reported in the literature has been found to contain a prfA* mutation nor to exhibit a PrfA* phenotype; all of the prfA* mutations reported have arisen spontaneously in laboratory media or as a result of chemical mutagenesis [30]–[33], [35], [37]. Moreover, field strains have been isolated containing missense mutations or small deletions within the coding region of the *prfA* gene that decrease or eliminate PrfA activity, indicating that PrfA activity is not required for optimal bacterial fitness outside of a host cell [64], [65] although a recent report indicates that some activity is required for efficient biofilm formation [66].

The link between carbon source utilization and PrfA regulation of *L. monocytogenes* virulence gene products has long been recognized but has remained poorly defined. Previous studies have demonstrated that when *L. monocytogenes* was grown in the presence of glucose or other carbon sources taken up by the phosphoenolpyruvate (PEP) transport system (PTS), the expression levels of PrfA-dependent genes were decreased [29], [67], [68]. Other studies have reported that over expression of prfA* on a multicopy plasmid in *L. monocytogenes* significantly impaired bacterial growth and glucose uptake in media where glucose was the main or sole carbon source [43], [44]. Although the isogenic prfA* strains used in this study did not exhibit the pronounced growth defects previously reported in the presence of glucose in monoculture, the addition of glucose to LB was found to exacerbate the competitive defect exhibited by the *prfA* G145S mutant in LB (Fig. 2B). While differences related to prfA* copy number may influence the ability of L. monocytogenes prfA* mutants to utilize glucose, the results in either case indicate that the presence of glucose decreases the fitness of *L. monocytogenes* when PrfA is activated.

In addition to the glucose-related growth defects reported for *L. monocytogenes* strains containing multicopy plasmid-encoded prfA*, it has also been reported that similar strains exhibited a subtle monoculture growth defect when grown in media where glycerol (a non-PTS carbon source) was the main carbon source [44]. In contrast to this finding, our data indicate that isogenic prfA* mutants were enhanced for glycerol utilization and exhibited a competitive advantage over wild type in the presence of glycerol (Fig. 2). While the competitive advantage was evident during the first three cycles of culture dilution and outgrowth, the wild type strain appeared to adapt to glycerol as a carbon source such that bacterial mutant to wild type ratios became stable after three cycles of growth (Fig. 2). Consistent with an adaptation of wild type *L. monocytogenes* to growth with glycerol, it was observed that after five cycles of dilution and outgrowth monocultures of wild type bacteria in LB glycerol reached the same cell densities as did the isogenic prfA* mutants but retained low levels of PrfA-dependent gene expression (J. Bruno, unpublished). L. monocytogenes prfA* strains thus appear to exist in a metabolic state that favors bacterial growth in glycerol but not glucose. The prfA* metabolic shift might thus enhance bacterial growth in the cytosol, where three carbon sugars have been suggested to be preferentially used for bacterial replication [69], [70].

In addition to the PrfA*-related metabolic shift of *L. monocytogenes* towards glycerol utilization, our data indicate that the competitive defects exhibited by prfA* mutants in broth culture were exacerbated under conditions of osmotic and/or acid stress (Fig. 3). Based on the reports of functional overlap of SigB and PrfA in regulation of *L. monocytogenes* gene expression [40], [53]–[60] and the fact that SigB is one of the most characterized stress response regulators of *L. monocytogenes* [52], it seemed logical to investigate whether the stress-associated fitness defects exhibited by the prfA* mutants were related to alterations in SigB function. Examination of the fitness of prfA* Δ*sigB* double mutant strains in BHI indicated that the double mutant exhibited a competitive defect that was approximately equivalent to the sum of the competitive defects exhibited by the prfA* and Δ*sigB* single mutants (Fig. 4). Thus, while constitutive PrfA activation appears to interfere with the stress response of *L. monocytogenes*, it does so independently of SigB. It is possible that PrfA activation may somehow interfere with the function of another general stress response factor, such as ClpC, whose expression has been shown previously to be influenced by PrfA activity [71], [72]. Alternatively, constitutive activation of PrfA may interfere with the expression or function of a factor(s) whose activity is directly involved in the repair of a stress associated cell injury.

In contrast to bacterial fitness in culture media, the need for down-regulation of PrfA activity does not appear to be required within the infected host. Experiments with either intravenously or intragastrically infected mice indicated that the prfA* mutants were more virulent than wild type *L. monocytogenes*. More bacteria were recovered from the livers and spleens of mice infected with prfA* mutant bacteria compared to those infected with wild type bacteria, and the mutant strains exhibited a competitive advantage in mixed infections (Figs. 5 and 6). One surprising finding was that despite an increased susceptibility to osmotic and acid stresses in culture media (Fig. 3), the prfA* mutants remained hyper-virulent following oral infection (Fig. 6). The GI tract presents *L. monocytogenes* with a variety of stresses, including acid and osmotic stress [49] as well as stress associated with mucous barriers [73]. Given that SigB contributes to the gastrointestinal survival of *L. monocytogenes* [57], [74] and that SigB function does not appear to be affected by constitutive PrfA activation (Fig. 4), prfA* mutants have the ability to respond to the stresses of the GI tract via SigB. In addition, the PrfA*-dependent increase in gene products that facilitate bacterial invasion (for example, InlA, InlB, LLO, ActA) [5], [18], [62], [75]–[79] and/or bile resistance (Bsh, BilE) [54], [55] may enhance intestinal translocation so as to counter balance any stress-associated defects.

In summary, the findings presented in this study emphasize the critical need for *L. monocytogenes* to regulate PrfA activity dependent on its environmental location. While experiments in broth culture indicate a competitive fitness defect for prfA* mutants, it remains possible that PrfA activation contributes to *L. monocytogenes* outside of mammalian infection, for example by promoting bacterial survival in the presence of lower eukaryotes or other soil dwellers. PrfA activation clearly enhances bacterial virulence in mammalian hosts, however the need for down modulation of PrfA activity in other settings might well be a reflection of the yin-yang nature of the *L. monocytogenes* saprophyte-pathogen balance.

## Materials and Methods

### Bacteria and culture media

The bacterial strains and plasmids used in this study are listed in Table 1. All *L. monocytogenes* strains used were derived from the 1/2a serotype 10403S *L. monocytogenes* strain, which is a streptomycin-resistant derivative of strain 10403 [80], [81]. The phenotypes reported for strains containing prfA* mutations were verified in independent isolates constructed by allelelic exchange and/or by phage transduction, and by comparison of different prfA* alleles (*prfA* G145S, *prfA* L140F, *prfA* Y63C). *L. monocytogenes* strains were grown in brain heart infusion (BHI) (Difco Laboratories, Detroit, MI) or Lysogeny Broth (LB) (Invitrogen Corp., Grand Island, NY). *Escherichia coli* strains were grown in LB. When appropriate, LB was supplemented with 55 mM of either glucose or glycerol. To decrease or increase medium acidity, BHI or LB was buffered to pH 7.4 with 100 mM of 3-(N-morpholino)propanesulfonic acid (MOPS) pH 7.4 (Sigma Chemical Co., St. Louis, MO) or to pH 5.5 with 100 mM of 2-(N-morpholino)ethanesulfonic acid (MES) pH 5.5 (Sigma), respectively. To increase medium osmolarity, BHI was supplemented with 5% sodium chloride (NaCl). The antibiotics (and concentrations) used in this study were: neomycin (5 µg/mL), chloramphenicol (10 µg/mL), erythromycin (1 µg/mL), and streptomycin (200 µg/mL).

**Table 1:** Bacterial strains and plasmids used in this study.

Strain	Description/Genotype	Designation	Reference
TOP10,SM10	*E. coli* strains for constructing recombinant plasmids		
NF-L100	10403S wild type		[80], [81]
NF-L890	NF-L100 Δ*prfA*		[33]
NF-L476	NF-L100 *actA-gus-plcB*		[89]
NF-L1124	NF-L100 *actA-gus-neo-plcB*	WT 10403S	[30]
NF-L1123	NF-L890 *actA-gus-neo-plcB*	Δ*prfA*	[30]
NF-L943	NF-L476 *prfA* G155S	*prfA* G155S	[32]
NF-L1177	NF-L1124 *prfA* G145S	*prfA* G145S	[41]
NF-L1166	NF-L1124 *prfA* L140F	*prfA* L140F	[41]
NF-L1006	NF-L476 tRNA^Arg^::pPL2	WT cam^R^	
NF-E1613	TOP10 with pTJA-57		
FSL A1-254	10403S Δ*sigB*	Δ*sigB*	[84]
NF-L1774	NF-L943 Δ*sigB*	*prfA* G155S Δ*sigB*	This study
NF-L1775	NF-L1177 Δ*sigB*	*prfA* G145S Δ*sigB*	This study
DP-L3903	10403S with Tn*917* insertion	WT erm^R^	[90]
NF-E1458	*E. coli* with HEL-913		[38]
NF-L1772	NF-L1124 *inlA* ^S192N,Y369S^	WT 10403S *inlA^m^*	This study
NF-L1773	NF-L1177 *inlA* ^S192N,Y369S^	*prfA* G145S *inlA^m^*	This study

Plasmid	Description/Genotype	Reference
pPL2	Site-specific phage integration vector	[87]
pKSV7	Temperature-sensitive integration vector for allelic exchanges	[85]
HEL-913	pKSV7::*inlA* ^S192N,Y369S^	[38]
pTJA-57	pKSV7::Δ*sigB*	[84]

### Construction of *L. monocytogenes* mutant strains via bacteriophage transduction

*L. monocytogenes* strain NF-L1775 (*prfA* G145S Δ*sigB*) was constructed by bacteriophage transduction as previously described [33], [82], [83]. Briefly, 10^7^–10^8^ PFU of *Listeria* phage U153 lysates [82] prepared from NF-L1177 (*prfA* G145S *actA-gus-neo-plcB*) [41] were mixed with 10^8^ CFU of mid-log FSL A1-254 (Δ*sigB*, a kind gift of Dr. Kathryn Boor, Cornell University, Ithaca, NY) [84]. The *prfA* G145S Δ*sigB* double mutant was confirmed to contain both the *prfA* G145S mutation and the downstream *actA-gus-neo-plcB* transcriptional fusion from the *prfA* G145S mutant [41] by isolating transductants that exhibited neomycin resistance and a blue colony appearance on BHI agar containing 50 µg/ml 5-bromo-4-chloro-3-indolyl-β-D-glucuronic acid (x-gluc).

### Construction of *L. monocytogenes* mutant strains via allelic exchange

*L. monocytogenes* strains NF-L1774 (*prfA* G155S Δ*sigB*), NF-L1772 (*inlA^m^*), and NF-L1773 (*prfA* G145S *inlA^m^*) were constructed using derivatives of the temperature-sensitive integration vector pKSV7 [85]. The *inlA^m^* mutation enhances the intestinal translocation of *L. monocytogenes* but has no impact on the outcome of intravenous infection [61]. Plasmid vector pTJA-57 (pKSV7::Δ*sigB*, kind gift of Dr. Kathryn Boor) [84], a pKSV7 derivative designed for the construction of Δ*sigB* mutations, was introduced into NF-L943 (*prfA* G155S) by electroporation as previously described [86]. Chromosomal integration of pTJA-57 and subsequent allelic exchange and plasmid curing were carried out as previously described [85]. The introduction of the Δ*sigB* mutation into the *prfA* G155S mutant background was confirmed by PCR amplification of the *sigB* open reading frame using primers LmsigB-15 and LmsigB-16 [84] (Table 2).

**Table 2:** Oligonucleotides used in this study.

Primer	Sequence (5′→3′)	Reference
LmsigB-15	AATATATTAATGAAAAGCAGGTGGAG	[84]
LmsigB-16	ATAAATTATTTGATTCAACTGCCTT	[84]
MARQ403	CAGATCTAGACCAAGTTACAA	[38]
MARQ408	CAGATCTAGAATAGTGACAGGTTGGCTAA	[38]

To facilitate the investigation of intragastric infections of mice, the *inlA^m^* (*inlA* ^S192N,Y369S^) mutation described by Wollert *et al.* [61] was introduced into a wild type 10403S strain (NF-L1124) and the *prfA* G145S mutant by electroporation, allelic exchange, and plasmid curing of the plasmid vector pHEL-913 (pKSV7-*inlA^m^*, a kind gift of Dr. Helene Marquis, Cornell University, Ithaca, NY) [38]. Strains NF-L1772 (*inlA^m^*) and NF-L1773 (*prfA* G145S, *inlA^m^*) were generated. The introduction of the *inlA^m^* mutation was confirmed by PCR amplification and DNA sequencing of the *inlA* open reading frame using primers MARQ403 and MARQ408 [38] (Table 2).

### Monoculture growth experiments

50 µL or 100 µL of an overnight culture grown in BHI were added to 12.5 mL or 25 mL, respectively, of fresh broth culture medium (a 1∶250 dilution) and incubated at 37°C with vigorous shaking and aeration. At specified time points, the optical density at 600 nm (OD_600_) of the culture was measured using a BioMate 3 UV-Vis Spectrophotometer (Thermo Fisher Scientific, Inc., Waltham, MA) and CFU/mL were determined by plating dilutions of a culture aliquot on BHI agar.

### Broth culture mixing experiments

The experimental design to assess the competitive index of a mixed bacterial broth culture is depicted in Figure S1. Briefly, equal amounts of bacteria from overnight cultures of wild type 10403S (reference strain) and a mutant or test strain grown in BHI were mixed at a 1∶250 dilution into a fresh BHI broth (or the indicated culture medium). To differentiate between the strains, the wild type 10403S reference strain contained the single copy integration plasmid pPL2 [87] to confer chloramphenicol resistance (designated WT cam^R^ strain in Table 1). Repeating cycles of culture growth and dilution (referred to as serial passages) were used to assess the competitive fitness of the test strain in comparison to wild type under a specific growth condition. Mixed cultures were incubated for 24 hours at 37°C with shaking and aeration and then diluted 1∶100 into fresh culture media and again grown for 24 hours at 37°C with shaking followed by a 1∶100 dilution. A total of nine cycles of growth and dilution (or 9 passages) were carried out in each serial-passage regime. Immediately prior to each dilution an aliquot of the mixed culture was removed, diluted, and plated onto BHI agar to obtain bacterial CFU counts. 150 of the resulting colonies were then patched onto BHI agar containing chloramphenicol to select for wild type cam^R^ bacteria. The competitive index (CI) value of the mixed culture was determined using the following equation: CI =  (test strain CFU)/(WT cam^R^ reference strain CFU). When the test strain was resistant to an antibiotic to which the WT cam^R^ reference strain was sensitive, aliquots were also plated on BHI agar containing the appropriate antibiotic. For example, the *prfA* G145S mutant is neomycin-resistant because of the *actA-gus-neo-plcB* transcriptional fusion it contains, but the WT cam^R^ strain is neomycin-sensitive, so aliquots from mixed cultures of the *prfA* G145S and WT cam^R^ strains were also plated on BHI agar containing neomycin. For graphic representation, the CI value of a mixed culture was plotted as a function of the mixed culture's dilution cycle number or passage number (passage #, P#), with ‘passage 0’ representing the initial mixture of two monocultures, ‘passage 1’ representing the mixed culture after the initial 24 hours of growth immediately prior to the first passage, ‘passage 2’ representing the mixed culture after the 24 hours of growth following the first passage and immediately prior to the second passage, etc. (Figure S1). pPL2 integration did not affect the competitive index of wild type 10403S in any growth condition as the WT cam^R^ strain never exhibited a competitive advantage nor disadvantage when mixed with the 10403S strain lacking the pPL2 inserted plasmid (CI values of ∼1 throughout the course of a mixing experiment) (Fig. 1C and J. Bruno, unpublished).

### Measurement of β-glucuronidase (GUS) activity

GUS activity was measured by an enzymatic assay as previously described [88]. Briefly, overnight cultures grown in BHI were diluted 1∶50 into fresh media and grown with shaking at 37°C. CFU/mL were measured at specified time points and two 500 µL culture aliquots were collected (for the *prfA* G145S and *prfA* L140F mutants, two 50 µL culture aliquots were collected because of their increased *actA-gus* expression) [41]. The aliquots were centrifuged (16,100×*g*) for 5 minutes, supernatants were removed, and one pellet from the two aliquots was suspended in 100 µL of ABT buffer (0.1 M potassium phosphate, pH 7.0, 0.1 M NaCl, 0.1% Triton) while the other was suspended in 1 mL of ABT buffer. Two 50 µL aliquots of each ABT bacterial suspension were pipetted into separate wells of a 96-well plate. 10 µL of 0.4 mg/mL of the GUS substrate 4-methylumbelliferyl-β-D-glucuronide (Sigma) were added to each 50 µL aliquot, and these mixtures were incubated at 37°C for 60 minutes. Substrate conversion was measured with a Barnstead/Turner Quantech FM109515 Fluorometer (Dubuque, IA). Units of GUS activity were calculated as previously described [88].

### Intravenous infections of mice

Animal procedures were IACUC approved by the UIC Animal Care Committee (Approval #09-153) and performed in the Biological Resources Laboratory at the University of Illinois at Chicago. Mid-log *L. monocytogenes* growing in BHI were washed, suspended, and diluted in PBS to reach a final concentration of 1×10^4^ CFU/mL or 1×10^5^ CFU/mL. 7–8 week old ND4 Swiss Webster mice (Harlan Laboratories, Inc., Madison, WI) were infected via tail vein injections with 200 µL of the bacterial suspensions, achieving an infectious dose (ID) of 2×10^3^ CFU or 2×10^4^ CFU, respectively. 24 or 48 hours post infection, the mice were sacrificed, and their livers and spleens were harvested. Each organ was placed in 5 mL of sterile Milli-Q water and homogenized with a Tissue Master-125 Watt Lab Homogenizer (Omni International, Marietta, GA). Homogenized tissues were diluted and plated on BHI agar containing streptomycin to determine CFU/organ.

### Oral infections of mice

Mid-log *L. monocytogenes* growing in BHI were washed, suspended, and diluted in PBS to reach a final concentration of 2.5×10^8^ CFU/mL or 2.5×10^10^ CFU/mL. 8–10 week old C57BL/6 mice (Harlan) were infected orally with 200 µL of the bacterial suspensions, achieving an ID of 5×10^7^ CFU or 5×10^9^ CFU, respectively. 72 hours post infection, mice were sacrificed, and their livers, spleens, stomachs, and intestines were harvested. The organs were homogenized and their bacterial loads were determined as described above.

## Supporting Information

Table S1 Logarithmic doubling times of *L. monocytogenes* strains under various conditions at 37C.
(DOC)

Figure S1 Experimental design of the broth culture mixing experiments. A detailed explanation is provided in Experimental Procedures.
(TIF)

Figure S2 Growth curves of *L. monocytogenes* strains in BHI at 37?C were determined by measuring CFUmL at the specified time points. The growth curves of wild type, *prfA* G155S, and *prfA *G145S *L. monocytogenes* in BHI are included Fig. 1B.
(TIF)
