# Supplementary material for: Constitutive Activation of PrfA Tilts the Balance of Listeria monocytogenes Fitness Towards Life within the Host versus Environmental Survival
Source: PLoS One. 2010 Dec 7;5(12):e15138. doi: 10.1371/journal.pone.0015138 (PMC2998416; doi:10.1371/journal.pone.0015138)
Supplement: Table S1 — Logarithmic doubling times of L. monocytogenes strains under various conditions at 37C. (DOC) [file pone.0015138.s001.doc]

| **^a^**Logarithmic doubling times are reported in minutes (± standard deviation).  CFU/mL from the **^b^**2 and 6 hour, **^c^**6 and 10 hour, **^d^**2 and 8 hour, **^e^**1 and 5 hour time points of monoculture growth experiments were used to determine the doubling times.  nd – not determined. | *prfA* G145S Δ*sigB* | *prfA* G155S Δ*sigB* | Δ*sigB* | Δ*prfA* | *prfA* L140F | *prfA* G155S | *prfA* G145S | WT | **Strain** |  | **Table S1. Logarithmic doubling times of *L. monocytogenes* strains under various conditions at 37°C.** |
| --- | --- | --- | --- | --- | --- | --- | --- | --- | --- | --- | --- |
|  | 44.6 | 39.81 | 41.92 | 37.77 (± 0.57) | 38.92 (± 1.95) | 40.4 (± 0.23) | 41.67 (± 1.46) | 36 (± 0.26) | **BHI^b^** | **Doubling Times^a^** |  |
|  | nd | nd | nd | nd | nd | nd | 44.77 | 38.36 | **LB^b^** |  |  |
|  | nd | nd | nd | nd | nd | nd | 37.98 | 31.23 | **LB + glu^b^** |  |  |
|  | nd | nd | nd | nd | nd | nd | 37.33 | 34.06 | **LB + gly^b^** |  |  |
|  | nd | nd | nd | nd | nd | 66.31 | 68.82 | 66.42 | **BHI +**  **5% NaCl^c^** |  |  |
|  | nd | nd | nd | nd | nd | nd | 66.74 | 66.54 | **BHI pH 5.5^d^** |  |  |
|  | nd | nd | nd | nd | nd | nd | 42.69 | 35.81 | **BHI pH 7.4^b^** |  |  |
|  | nd | nd | nd | nd | 56.7 (± 4.08) | 66.45 (± 7.18) | 61.99 (± 9.18) | 72.9 (± 10.48) | **Intra-**  **cellular^e^** |  |  |
