# Supplementary figures and images for: Constitutive Activation of PrfA Tilts the Balance of Listeria monocytogenes Fitness Towards Life within the Host versus Environmental Survival
Source: PLoS One. 2010 Dec 7;5(12):e15138. doi: 10.1371/journal.pone.0015138 (PMC2998416; doi:10.1371/journal.pone.0015138)

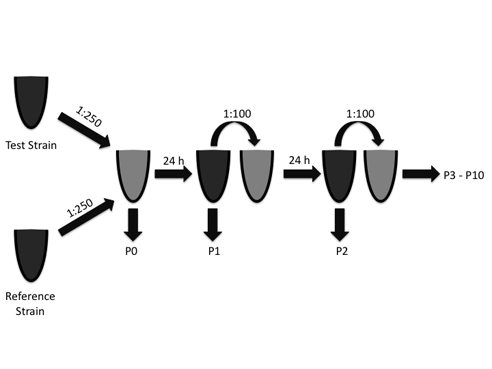

Supplement: Figure S1 — Experimental design of the broth culture mixing experiments. A detailed explanation is provided in Experimental Procedures. (TIF) [file pone.0015138.s002.tif]

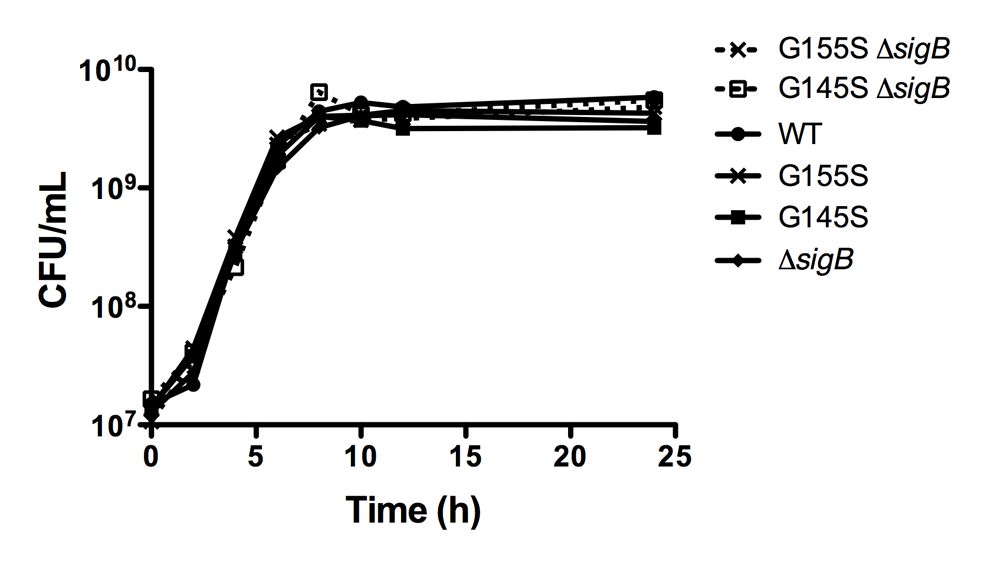

Supplement: Figure S2 — Growth curves of L. monocytogenes strains in BHI at 37?C were determined by measuring CFUmL at the specified time points. The growth curves of wild type, prfA G155S, and prfA G145S L. monocytogenes in BHI are included Fig. 1B. (TIF) [file pone.0015138.s003.tif]
